# Nighttime assaults: using a national emergency department monitoring system to predict occurrence, target prevention and plan services

**DOI:** 10.1186/1471-2458-12-746

**Published:** 2012-09-06

**Authors:** Mark A Bellis, Nicola Leckenby, Karen Hughes, Chris Luke, Sacha Wyke, Zara Quigg

**Affiliations:** 1 Centre for Public Health, Liverpool John Moores University, 15-21 Webster Street, Liverpool, L3 2ET, UK; 2 Cork University Hospital, Wilton, Cork, Ireland

**Keywords:** Violence, Assaults, Emergency department, Nighttime, Deprivation, Monitoring

## Abstract

**Background:**

Emergency department (ED) data have the potential to provide critical intelligence on when violence is most likely to occur and the characteristics of those who suffer the greatest health impacts. We use a national experimental ED monitoring system to examine how it could target violence prevention interventions towards at risk communities and optimise acute responses to calendar, holiday and other celebration-related changes in nighttime assaults.

**Methods:**

A cross-sectional examination of nighttime assault presentations (6.01 pm to 6.00 am; n = 330,172) over a three-year period (31^st^ March 2008 to 30^th^ March 2011) to English EDs analysing changes by weekday, month, holidays, major sporting events, and demographics of those presenting.

**Results:**

Males are at greater risk of assault presentation (adjusted odds ratio [AOR] 3.14, 95% confidence intervals [CIs] 3.11-3.16; P < 0.001); with male:female ratios increasing on more violent nights. Risks peak at age 18 years. Deprived individuals have greater risks of presenting across all ages (AOR 3.87, 95% CIs 3.82-3.92; P < 0.001). Proportions of assaults from deprived communities increase midweek. Female presentations in affluent areas peak aged 20 years. By age 13, females from deprived communities exceed this peak. Presentations peak on Friday and Saturday nights and the eves of public holidays; the largest peak is on New Year’s Eve. Assaults increase over summer with a nadir in January. Impacts of annual celebrations without holidays vary. Some (Halloween, Guy Fawkes and St Patrick’s nights) see increased assaults while others (St George’s and Valentine’s Day nights) do not. Home nation World Cup football matches are associated with nearly a three times increase in midweek assault presentation. Other football and rugby events examined show no impact. The 2008 Olympics saw assaults fall. The overall calendar model strongly predicts observed presentations (R^2^ = 0.918; P < 0.001).

**Conclusions:**

To date, the role of ED data has focused on helping target nightlife police activity. Its utility is much greater; capable of targeting and evaluating multi-agency life course approaches to violence prevention and optimising frontline resources. National ED data are critical for fully engaging health services in the prevention of violence.

## Background

Globally, interpersonal violence is a major threat to health [1]. It causes over half a million deaths each year and is a leading cause of mortality and morbidity in young people [2]. Treating the physical and mental health impacts of violence imposes significant costs on health services [3]. The burden of violence falls disproportionately on lower and middle income countries [1], yet even in England and Wales there are estimated to be over two million incidents of violence against adults and at least half a million more against children each year [4], resulting in annual costs to the health service of over £2 billion [5]. Assaults on health service staff alone cost around £60 million per year [6]. When broader economic, criminal justice, and social impacts are included violence is estimated to cost the country £24 billion annually [5]. Across England surveys suggest that 67% of assaults occur at nighttime [4]; often following alcohol consumption. Nearly half (44%) of all assaults are alcohol-related (equivalent to almost one million annually) and one in five takes place near a bar, pub or other licensed drinking venue [4].

The immediate nature of treatment required for assaults, and their focus in nighttime hours, means resulting injuries impact particularly on emergency department (ED) services. This relationship has resulted in ED data being recognised by the World Health Organization as key intelligence for the development, implementation, and evaluation of violence prevention initiatives [7,8]. Importantly, in both the UK and elsewhere, a large proportion of assaults resulting in ED treatment are not reported to police [9-11]. Consequently, the intelligence EDs can add to existing criminal justice systems has led to the development of local and regional ED violence surveillance systems in some countries (e.g., UK [11,12], Jamaica [13], Colombia, El Salvador and Nicaragua [14]). Assault location, which can be recorded when individuals present in the ED, has been used to target police and licensing authority enforcement activity on assault ‘hot spots’ such as problem bars with some success in reducing violence [11,12]. However, the utility of robust ED data in violence prevention is not limited to targeting judicial activity. Numerous studies identify effective roles for health care and public health services in violence prevention; including through identifying and supporting victims [15], and through commissioning and delivering selective (e.g., perinatal support, parenting programmes, and pre-school enrichment) and indicated (e.g., cognitive behavioural therapy and multisystemic therapy) prevention programmes [16-20]. ED data could be used to target such interventions at those communities and individuals most at risk of violence so long as residential data are collected [21]. Moreover, retrospective ED data may be used to predict and prepare for future pressures on services created by the economy (e.g., recession [22]), calendar events (e.g., new national holidays [23]), festivals and sporting occasions (e.g., Olympics [24]) or changes in policy (e.g., national alcohol licensing [25]). With often limited health resources, such intelligence should inform more effective and economic service planning; helping ensure services are better directed to when and where they are needed. Nevertheless, globally the national ED data systems required to examine these issues on a routine basis are almost entirely absent [26,27].

England began an experimental national ED data collection system in 2007 [28]. These data are used here to examine the demographic characteristics of those presenting for nighttime assaults and the impact of temporal events (day of week, bank holidays, seasons, special events) on nighttime ED assault attendances in different communities. We examine how such data could be used nationally and locally to minimise assaults through: targeting prevention interventions at those communities and individuals most affected by violence; better planning of, and preparation for, holidays and events; and optimising the deployment of front line resources.

## Methods

Since 1989, the Hospital Episodes Statistics (HES) service has recorded all episodes of inpatient care at National Health Service hospitals across England, including private patients. In 2007, the HES system was expanded to record a basic dataset of all presentations in accident and emergency services (HES A&E), including major EDs, single speciality EDs, walk-in centres and minor injury units. The dataset records age, sex, time, date of attendance, and postcode of attendee’s residence. It classifies attendance into nine broad categories (road traffic accident, assault, deliberate self-harm, sports injury, firework injury, other accident, brought in dead, other than above and not known) [28]. Data also distinguish first time attendances from follow-ups. Data are collated throughout the care pathway by administrative and medical staff in emergency services and are submitted, from patient administrative systems, by clinical service providers to a national Secondary Uses Service [29] for planning, monitoring and other research purposes. The content and access to the HES service are managed by the Health and Social Care Information Centre [30]. Currently, 187 clinical service providers contribute any data to the HES A&E system, compared with 327 providers contributing to the QMAE system (Quarterly Monitoring of Accident and Emergency data; a simple national count of solely number of attendances at any emergency service). However, all 150 providers with major EDs contribute data (covering 199 major EDs, including 9 children’s EDs) with non-contributors accounted for by smaller or specialist emergency services (e.g., walk in centres and minor injury units) which are typically closed over night. Despite all providers with major EDs contributing to the HES A&E system, data are incomplete. Providers with major EDs submitted data on a total of 14,821,225 attendances for any reason in 2010/11, compared with 15,712,068 attendances reported through QMAE (94.3% coverage). However, data from nine providers included no cases of violence, indicating that data coding issues remain in this experimental data system.

For the period 31^st^ March 2008 to 31^st^ March 2011 all first time presentations (i.e., excluding follow up presentations) for assaults were extracted from the HES A&E data (n = 526,687). The HES system automatically maps postcode of residence to lower super output areas (LSOAs; geographical areas with a population mean of 1500 designed to standardise reporting of small area statistics in England and Wales [31]). Each LSOA has an average measure of deprivation routinely calculated across residents based here on the 2010 Index of Multiple Deprivation (IMD), a composite measure that includes 38 indicators relating to health, economic and educational status [32]. We assigned presentations to a national quintile of deprivation based on the IMD values of their LSOA [33]. While data were available for ED presentations at any time of day, we limited analysis to night times (6:01 pm-6:00 am; n = 330,172; 62.7% of all recorded attendances), when EDs are practically the only treatment option for those requiring immediate attention and thus data are not confounded by choice of different treatment services. In the final HES A&E data set 98.8% of nighttime assault presentations were from major EDs and 1.2% from other data providers (e.g. walk in and minor injury units).

For any day, the 12 hour nighttime period was assigned to the date relating to the first six hours (e.g., attendances occurring between 6:01 pm on 29/09/2009 and 6:00 am 30/09/2009 = nighttime attendances for 29/09/2009 [11]). As a result complete data were only available between 31^st^ March 2008 and 30^th^ March 2011 and analyses were limited to this period. Where denominator populations were required we used 2009 mid-year populations by age, sex, and deprivation; with deprivation again being based on the LSOA of residence. For temporal analyses, discrete calendar dates and events were chosen on the basis of whether they were nationally stipulated public holidays, established dates commemorating well known individuals or events on a national basis or, sporting events of national interest (Table 1). All data provided through HES A&E are anonymised but the system can assign individuals with a unique identifier. For our sample, while some individuals presented for nighttime assaults more than once in the three-year period (number of times presenting; 1, 93.3%; 2, 5.8%; >2 0.9%) analyses focus on number of presentations, not individuals.

**Table 1:** Calendar and sporting events included in assault presentation analyses

**Event**	**Details**
*Calendar*	
Year^1^	2008/09-2010/11
Month of year	January to December
Day of week	Sunday to Saturday
English bank holidays	Any national public holiday
England bank holiday eves	Day before a public holiday
New Year’s eve and day	31st December and 1st January
Christmas eve and day	24th and 25th December
Last and first week of month and last and first two days of month	Examining payday effects e.g., celebrating receiving a monthly wage
St George's day	23rd April
St Patrick’s day	17th March
Halloween	31st October
Guy Fawkes (Bonfire) night	5th November
Valentine’s day	14th February
*Sporting Events*	
Football Association Cup	Annual finals
UEFA Champions League Final	Union of European Football Associations Champions League final
Football World Cup 2010	Includes only England matches
Rugby Six nations	Includes only England matches annually
Summer Olympic Games 2008	8th-24th August 2008

^1^ Years run from 31^st^ March to 30^th^ March to account for time-shifted data.

Data were extracted for analysis in Predictive Analytics Software (PASW®) Version 18. Analysis used ANOVA for direct comparisons between different daily assault attendances. Binary logistic regression (LR) was used for calculation of adjusted odds of attendance by demography with non-attendees calculated by age, sex, and deprivation specific subtraction of assault presentations from national matching population numbers. LR was used as the dependent variable was binary and the categorical independent variables fulfilled the criteria for such modelling [34]. Generalised linear modelling (GLM) is a robust technique for modelling count data (e.g., here presentations per day) over a fixed time period [35] and was employed here to examine independent impacts of calendar days, holidays, and sporting events on nighttime attendance levels. Although a large data set, over the three-year period some calendar events occur just one day a year (e.g., New Year’s Eve). Thus, to reflect the range of reliability, confidence intervals are presented for both basic descriptive statistics (Table 2) and modelled relationships (Table 3). Deprivation rate ratios (DRRs) were calculated as the ratio of the most deprived quintile (IMD5) to the most affluent (IMD1) for gender specific rates in single year of age categories up to age 75 years. With data conforming to normality, comparisons between deprivation rate ratios for males and females used a paired (by age) sample T test.

**Table 2:** **Variations with calendar event in average numbers**^**$**^** of per evening assault presentations across English emergency department services**

**Calendar events**			**All attendances**				**Males**				**Females**			
				**95% CI**				**95% CI**				**95% CI**		
		**Days**	**Mean**	**Low**	**Up**	**Sig**	**Mean**	**Low**	**Up**	**Sig**	**Mean**	**Low**	**Up**	**Sig**
All days		1095	301.4	292.1	310.8		227.3	219.7	234.9		72.6	70.8	74.4	
Holidays	No holiday	1072	301.7	292.1	311.2	0.946	227.7	219.9	235.4	0.869	72.5	70.7	74.3	0.940
	Bank holiday	18	287.7	236.6	338.8		209.1	168.0	250.2		77.0	66.8	87.2	
	Christmas	3	264.0	154.8	373.2		189.7	95.8	283.6		73.3	58.2	88.5	
	New Year	3	278.0	161.6	394.4		205.0	88.8	321.2		72.7	66.4	78.9	
Holiday	No holiday	1071	296.0	287.0	305.0	<0.001	222.9	215.6	230.2	<0.001	71.6	69.9	73.4	<0.001
Eves	Bank holiday	18	447.1	390.0	504.1		340.9	293.5	388.4		102.2	92.2	112.3	
	Christmas	3	485.3	422.2	548.5		399.7	323.2	476.0		83.3	54.5	112.1	
	New Year	3	1196.3	975.0	1417.7		956.7	765.1	1148.2		231.0	185.4	276.6	
Day^i^	Sunday	142	230.2	221.8	238.6	<0.001	166.2	159.6	172.9	<0.001	62.9	60.7	65.2	<0.001
	Monday	156	198.6	193.2	204.0		143.6	139.5	147.7		54.2	52.3	56.0	
	Tuesday	157	186.1	181.0	191.3		132.9	128.9	136.8		52.5	50.8	54.2	
	Wednesday	155	197.8	190.7	204.8		143.6	137.7	149.5		53.4	51.6	55.2	
	Thursday	151	227.2	221.2	233.2		170.1	165.3	174.9		56.0	54.3	57.8	
	Friday	154	503.1	491.8	514.3		393.2	383.7	402.6		107.1	104.5	109.6	
	Saturday	156	523.1	509.1	537.0		406.0	395.0	417.0		114.3	111.0	117.6	
Calendar	Jan	92	246.0	219.7	272.3	<0.001	184.9	163.5	206.4	0.003	59.9	54.9	64.9	<0.001
Month^i^	Feb	84	280.8	249.0	312.6		212.3	186.6	238.1		66.9	60.8	73.0	
	Mar	93	267.1	240.2	294.0		200.8	178.8	222.8		65.6	60.5	70.7	
	Apr	86	309.7	277.2	342.2		231.9	205.8	258.1		75.5	69.1	81.8	
	May	87	334.0	300.1	367.8		251.3	223.4	279.1		80.3	74.2	86.3	
	Jun	90	321.2	287.5	354.9		238.8	211.7	266.0		80.5	74.0	87.0	
	Jul	93	319.2	287.8	350.6		238.5	213.1	263.9		79.3	73.1	85.5	
	Aug	90	322.7	293.2	352.3		241.2	217.0	265.4		80.2	74.6	85.8	
	Sep	90	297.5	266.0	329.1		224.8	199.0	250.7		71.4	65.6	77.3	
	Oct	93	312.0	276.4	347.6		238.5	209.5	267.4		72.4	65.6	79.2	
	Nov	90	274.0	244.2	303.8		205.8	181.6	230.0		67.1	61.3	72.9	
	Dec	83	266.7	236.6	296.7		205.7	180.6	230.8		59.6	54.4	64.9	
Change of	Neither	935	296.6	287.0	306.1	0.205	223.6	215.8	231.3	0.189	71.5	69.7	73.8	0.257
month^i,ii^	First 2 days	69	269.7	237.9	301.6		200.1	174.5	225.7		68.4	61.9	74.9	
	Last 2 days	67	314.7	271.7	357.8		236.9	201.8	272.0		76.4	68.4	84.5	
Change of	Neither	599	294.2	282.3	306.1	0.288	222.0	212.3	231.7	0.295	70.8	68.5	73.0	0.203
month^i,ii^	First week	246	288.1	269.9	306.2		215.7	201.2	230.3		70.9	67.2	74.6	
	Last week	226	309.1	288.2	329.1		233.0	216.0	250.1		74.7	70.7	78.6	
Year^iii^	2008/09	366	285.5	270.1	300.8	0.060	215.0	202.5	227.5	0.078	69.8	66.9	72.8	0.067
	2009/10	365	309.1	292.1	326.0		233.1	219.4	246.9		73.0	69.8	76.2	
	2010/11	364	309.8	293.5	326.2		233.9	220.6	247.2		74.9	71.8	78.1	

^$^National emergency department data are not complete for England and therefore figures do not represent national totals - see Methods. Statistics use analysis of variance. ^i^Variables exclude banks holiday and Christmas eves. ^ii^Change of month variables mark the first and last two days and first and last week in each month. ^iii^Years run 31^st^ March to 30^th^ March to account for time-shifted data.

**Table 3:** Generalised linear model examining independent impacts of calendar events on mean numbers of assault presentations per night to emergency department services in England

**Variable**		**Slope (B)**	**95%CIs**		**P**
Months	January	−31.22	−44.88	−17.56	<0.001
	February	13.64	−0.69	27.98	0.062
	March	6.14	−7.76	20.03	0.387
	April	41.02	27.18	54.86	<0.001
	May	52.44	38.61	66.27	<0.001
	June	46.99	33.04	60.93	<0.001
	July	51.03	37.35	64.70	<0.001
	August	59.88	45.53	74.24	<0.001
	September	36.54	22.75	50.32	<0.001
	October	31.11	17.32	44.90	<0.001
	November	6.43	−7.47	20.34	0.365
	December (Ref)				
Weekends &	Fri-Sat	303.13	296.84	309.42	<0.001
Holidays	Bank Holiday Eves Sun-Thur	242.74	221.55	263.94	<0.001
	New Year’s Eve Sun-Thur	970.35	904.72	1035.98	<0.001
	Bank Holiday Eves Fri-Sat	275.85	183.57	368.13	<0.001
	New Year’s Eve Fri-Sat	1107.85	1015.57	1200.13	<0.001
	Sun-Thur (Ref)				
^i^Sporting	FA Cup final	53.62	−0.45	107.68	0.052
Events	Six Nations Rugby	7.65	−18.41	33.72	0.565
	World Cup Football England	298.05	251.10	344.99	<0.001
	Olympics	−38.07	−62.70	−13.44	0.002
	UEFA Champions final	32.03	−21.86	85.92	0.244
^i^Celebrations	St Patrick's day	65.38	11.49	119.26	0.017
	Halloween	191.99	138.10	245.88	<0.001
	Valentine's day	−12.61	−68.61	43.40	0.659
	Guy Fawkes night	96.37	42.50	150.24	<0.001
	St George's day	7.45	−46.43	61.33	0.786

^i^ Sporting events and celebrations are all entered into the generalised linear model as separate binary variables. Reference categories have been omitted. Data cover the time period 31st March 2008 to 30th March 2011 (see Methods). 95%CI = 95% confidence intervals. Full details of calendar events, sporting events and celebrations are given in Table 1.

The HES data system is specifically compiled in order to be used for planning and research purposes [30]. The Centre for Public Health is compliant with the HES Protocol [36] (which covers data access and sharing issues) under the terms and conditions of which it undertakes work on HES data relating to the epidemiology of violence and alcohol and disseminates such information. Consequently, further ethical approval for analyses on this existing data system was not required.

## Results

Overall, 75.8% of presentations were male with 48.7% of all nighttime assault presentations falling on Friday and Saturday nights. Age at presentation peaked in teenagers and younger adults (under 15 years, 4.0%; 15–24 years, 45.9%; 25–34 years, 23.8%; 35–54 years, 22.9%; 55 years or over, 3.4%) with the lowest levels of nighttime presentations in those under 15 or over 54 years.

### Weekends and holidays

Consistent with findings from other ED and criminal justice studies [11,37,38], nighttime assaults here show strong weekly peaks in presentations on Friday and Saturday nights (Table 2). England has a series of set one-day public holidays each year (see Table 1). The nighttimes of these holidays were not associated with an increase in assault presentations. However, their eves (nights before the holiday) showed significant increases in assaults (Table 2). New Year’s Eve showed a greater increase in assaults than that associated with other bank holidays, while the increase seen on Christmas Eve was consistent with increases with bank holidays generally (Table 2). Consequently Christmas Eve, but not New Year’s Eve, was incorporated with other bank holidays in further analyses, and nights of the week were categorised as Sunday-Thursday and Friday-Saturday. Using GLM to test the independent significance of calendar effect, the impacts of Friday-Saturday, bank holiday eves, and the even greater impact of New Year’s Eve on increasing assaults were maintained (Table 3).

### Annual and monthly effects

There was no significant impact of year (Table 2) and consequently this was eliminated from further analyses. Bivariate analyses identified an increase in nighttime assault presentations over the summer months (Table 2). End of month effects (the impact of being paid at the end of month) were not apparent either when examining the first or last two days or the first or last week of months, and therefore were eliminated from further analyses (Table 2). Using GLM, monthly assaults showed an overall nadir in January with a rise over the summer period, peaking in August (Table 3).

### Celebrations without a public holiday

**Figure 1:**
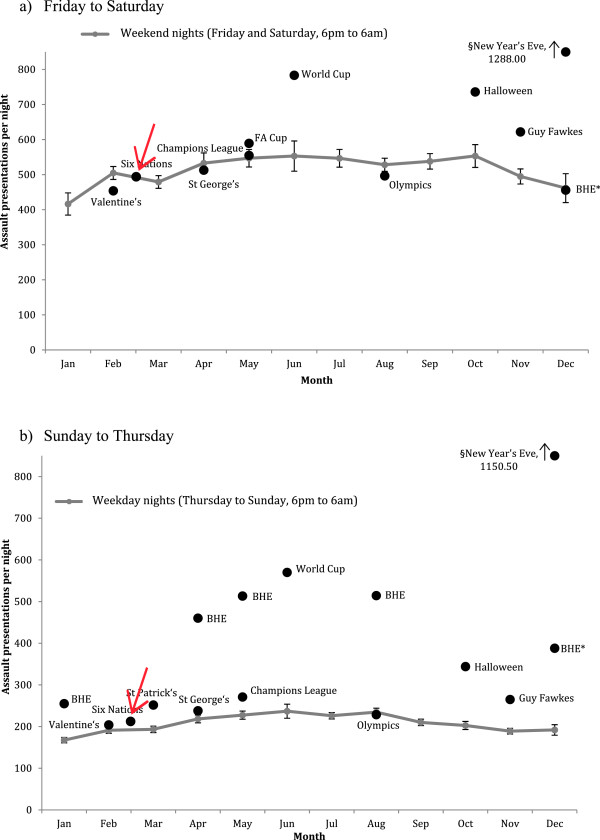
**Mean assault presentations per night by month and for holidays, sporting events, or other celebrations; a) Friday and Saturday nights; b) Sunday to Thursday nights.** 95% confidence intervals are presented for each month only. For statistical significance of differences see Table 3. Full descriptions of the holidays, sporting events, and celebrations included are given in Table 1. BHE = Bank holiday eve; § New Year’s Eve has mean value provided as it is outside the y axis scale.

A number of other national celebrations (not linked to holidays) were included in the GLM analyses (Table 1). Halloween, Guy Fawkes and St Patrick’s nights were all associated with significantly increased levels of assault presentations (Table 3). However, St George’s Day and Valentine’s Day nights had no significant impact. Figure 1 shows how holiday eves and some non-holiday related celebrations increased assaults within certain months of the year, when they occurred on (a) Fridays-Saturdays or (b) Sundays-Thursdays.

### Sporting events

Figures 1a and 1b also identify the relationship between key sporting events and increased assaults. Of those examined, the greatest increase in assault presentations was associated with national team (England) matches in the football 2010 World Cup; with presentations nearly tripling when matches occurred on Sunday-Thursday evenings (June; Figure 1a). Finals of the Football Association Cup, the Union of European Football Associations Champions League, and Rugby Six Nations England matches showed no significant impact (Table 3). However, the 2008 Olympics were associated with a small but significant fall in assault presentations and this effect was maintained even when GLM analysis was limited to 2008 (*X*^2^ = 5.733; P < 0.05). The overall calendar model (Table 3) including night of the week, holiday eves, celebrations, and sporting events was a strong predictor of observed values (observed *v* modelled estimates; R^2^ = 0.918; P < 0.001).

### Sex and deprivation effects

**Figure 2:**
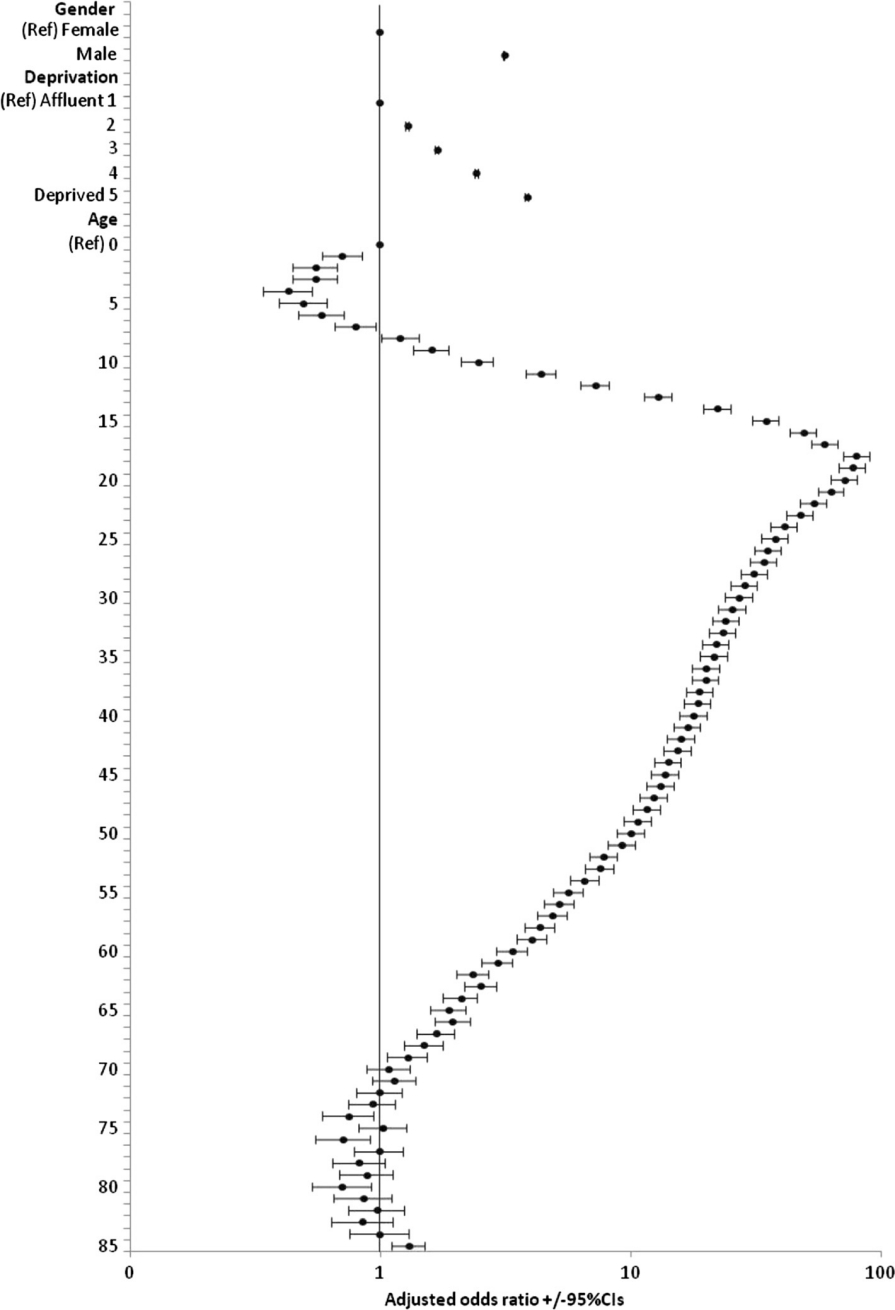
**Logistic regression model for risk of nighttime assault presentation by age, sex and quintile of deprivation.** Model uses backward conditional logistic regression. Age, sex and deprivation quintiles variables all made highly significant contributions to the model; Wald statistic = 220912.72, 76813.16, 69763.54 respectively; P < 0.001 for each variable.

Numbers of assault presentations were significantly higher for males than for females (Table 2; t = 50.84, P < 0.001). Male to female assaults ratios were highest on peak days for assaults; increasing from a mean of 2.76 (95% CIs 2.72-2.80) on Sunday-Thursday nights to 3.64 (95% CIs 3.59-3.69) on Friday-Saturday nights (t = 24.40; P < 0.001), and further to 4.14 (95% CIs 3.86-4.43) on New Year’s Eve. Examining assaults by deprivation identified that the proportion of assault presentations on Sunday-Thursday nights increased with deprivation (most affluent to most deprived quintile, 46.02%, 47.21%, 49.49%, 51.41% & 54.23%; *X*_trend_^2^ = 1065.22, P < 0.001). LR analysis examined the individual contribution of age, sex, and deprivation to likelihood of presenting in the ED for a nighttime assault (Figure 2). Risks peaked strongly at age 18 years. Odds of assault presentation were more than three times higher for males (3.14, 95% CIs 3.11-3.16; P < 0.001) than for females and nearly four times greater for the most deprived quintile than the most affluent (3.87, 95% CIs 3.82-3.92; P < 0.001).

**Figure 3:**
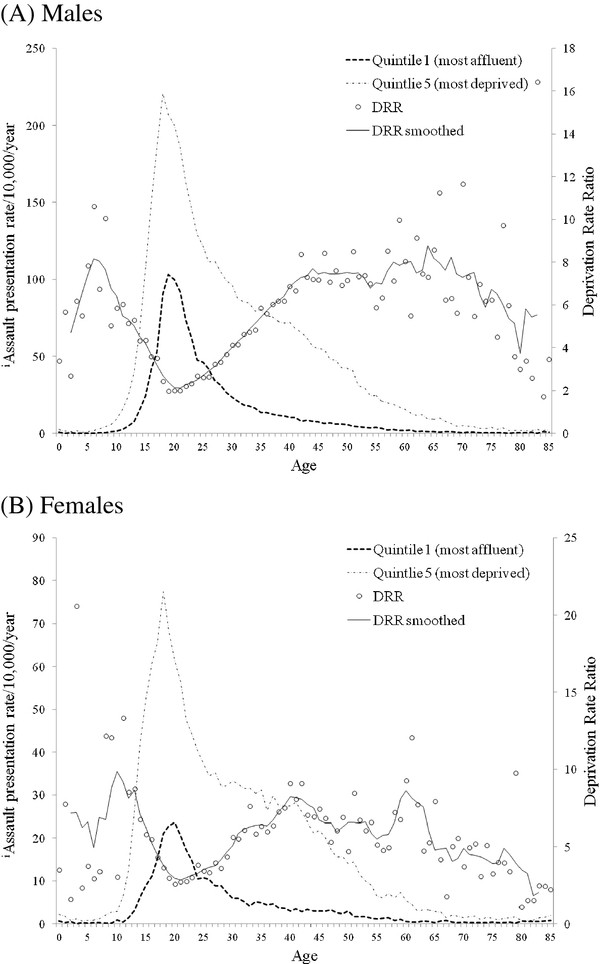
**Affluent and deprived quintile assault presentation rates**^**i**^** and deprivation rate ratios by age.** DRR = deprivation rate ratio. DRR smoothed is calculated as a five year rolling average. ^i^Not all ED services currently report violence data to the national database and therefore these are presented only for comparative purposes.

In order to examine how the impact of deprivation varies with age, deprivation rate ratios (DRRs; rate in most deprived quintile/most affluent quintile) were calculated (Figure 3a,b). DRRs in males showed a pre-pubescent peak around age six years, a nadir at 21 years then an increase to a post-adolescence plateau from approximately 45 years (Figure 3a). Variations in DRRs in females were similar to males. However, both the pre-pubescent rise and post-adolescence plateau were less well defined than for males (Figure 3b). Year wise paired comparison of DRRs between males and females identified no significant difference (t = 0.688, P = 0.493).

## Discussion

We have demonstrated that a national ED monitoring system can usefully identify individual and community risk factors for assault and changes in service pressures with calendar, celebration, and sporting events. Routine analyses of assault data often uses police recorded crime to examine the impact of calendar events on assaults. However, such data can be confounded by both levels of police activity (number of individuals working in any area), policing policy (e.g., which violent events warrant warning and which arrests) and where they take place (e.g., detection of assaults in public vs. private spaces). ED data are not directly impacted by such confounders, provide a measure of health harms relating to nighttime assaults and include events that are not reported to police [11].

Using such ED data this study identifies that nights preceding work-free days see more than double levels of assaults presentations (Figure 1a, b). Assault levels peak in summer months falling to a low in January (Figure 1a,b; Table 3), when alcohol consumption can also reach its nadir [39]. Although violence has been linked with warmer weather [40], a concentration of individuals’ personal holidays in the summer period may also be a contributing factor despite many individuals holidaying abroad [41]. Constraints that employment places on the length of nights out and alcohol consumption are removed not only by holidays but also by unemployment [42]. Thus, the most deprived communities showed the highest assault rates and a greater proportion of assaults on Sunday-Thursday nights; consistent with more individuals having no employment pressures midweek. Further, while deprived and affluent males both showed peaks in assaults rates in their late teens, rates reduced more rapidly in the most affluent (Figure 3a). Movement into employment in post-adolescence can reduce excessive alcohol use [43] – although how this impacts on exposure to violence is less well studied. We also identified deprivation-related differences in assault presentation at early ages. Critically, by age 15 years males in the most deprived quintile had exceeded the peak presentation level achieved in the most affluent quintile at age 19 years (Figure 3a). Worse, for females assault presentations in the affluent quintile peaked at age 20 years and rates in the most deprived quintile exceeded this peak by age 13 years (Figure 3b).

Globally, social inequality, poverty and youth unemployment have been associated with increased violence [44] and even rioting in some countries, including the UK [45]. Internationally, attention has focused on both immediate policing measures to prevent further violence and, increasingly, the need for longer-term multidisciplinary life course approaches to improve young people’s prospects and reduce their overall propensity for violence [1,17,46,47]. On the former, some local ED data systems have already been used to record assault location and inform the targeting of police activity [11-13] often in nightlife areas. On the latter however, potential roles for ED data remain largely underdeveloped. Results presented here identify a much earlier escalation in violence in the poorest communities and a peak at a much higher level (Figure 3a,b). Early life exposure to violence represents a direct risk to children’s immediate and long-term physical and mental health [48,49]; in some circumstances resulting in permanent disability. Moreover, such exposure also leaves individuals more likely to engage in violence later in adolescence and adulthood [48,50]. Early life exposure to assaults can be reduced through parental support, pre-school enrichment, and social development programmes [16-19]. Several of these programmes, such as Nurse Family Partnerships [16], have already begun to be scaled up in a number of countries including Canada [51], Australia [52] and England [53]. Here we have identified how a national ED data system can provide a benchmark; identifying areas most in need of such interventions.

As well as a role in targeted long-term prevention, we have shown that a national ED data system identifies peaks and troughs in violence that are strongly associated with events such as celebrations and sporting events. In England Halloween, Guy Fawkes Night and St Patrick’s Day are now heavily commercialised events with themed alcohol promotions, organised public events (such as club nights and bar crawls) and private parties. All three were associated with significant increases in assaults (Figure 1a,b; Table 3). However, Valentine’s Day and St George’s Day showed no significant increases. Sporting events also varied in impact on assault presentations. Presentations increased dramatically on nights when the national team played in the World Cup but not with other football or rugby fixtures (Figure 1a,b; Table 3). The association between sport and public violence has been examined elsewhere [54,55]. However, this study identifies how ED data can measure the impacts of violence beyond that typically observed around city centres and gatherings such as sporting events. Thus, broadcast access to the Beijing Olympics was associated with a small but significant reduction in overall assault presentations in England (Table 3). While understanding such patterns exposes expected pressures on ED departments, they are also pertinent to other frontline services such as ambulance and police. Currently, there is little information on how well emergency staffing levels are attuned to demand and national ED data, with local intelligence, could help inform the efficient distribution of staff and other resources on a calendar basis.

Our analysis only examined public holidays, national celebrations, and some major national and international sporting events. In planning holidays and events nationally more thought should be given to how selection of specific times, days, and months could be used to minimise any resultant increase in violence. Moreover, health and other agencies should consider such intelligence when timing campaigns to reduce binge drinking and related violence, stipulating license requirements, and enforcing critical legislation (for example, no sales of alcohol to those underage or already drunk).

The ability of ED data to provide intelligence on nighttime assaults relies on individuals reporting violence as the reason for their presentation. Such reporting may be affected by issues of confidentiality. More work is required on protecting confidentiality by establishing optimal levels of data access for different organisations and at different geographical levels [56]. While some local ED systems in England collect and share information on assault location, few share information on residence; despite this being routinely collected in the ED services. This combination of data is urgently needed to understand trends in and relationships between public (e.g., city centre) and private space (e.g., homes) violence. Together, these data would enable an effective multiagency response both nationally and locally. However, even the experimental data utilised here exposes some important gaps in our understanding of nighttime violence. Thus, some events are violence promoters (such as St Patrick’s Day and England games in the football World Cup), while others are nonbelligerent (such as St George’s Day and the Rugby Six Nations) or perhaps even protective (for example, the Olympics). The relative impact of different holidays and events may vary with locality and nation. For example in Cardiff, Wales (where rugby is often considered to be the national sport), international rugby matches involving the Welsh team have been associated with increased ED assault attendances [55]. Research is needed to understand the factors protecting relatively peaceful celebrations, and the roles commercialisation and linkage with alcohol promotions play in coupling celebrations with violence.

This study has a number of important limitations. English national HES A&E data are still incomplete. Although a full audit of data quality is not available, comparison with the QMAE suggests HES A&E represent 74% of all presentations regardless of cause [28]. HES A&E though covers all major EDs with much missing data arising from other emergency service providers such as walk in centres, which only accounted for 1.2% of nighttime assault presentations in this dataset. However, although emergency services are the principal resource for urgent assault treatment at night not all individuals assaulted, or even requiring treatment will present to them. England also has a general practice on call system where doctors can be asked to attend individuals’ places of residence. Further, injured individuals may also attempt to treat themselves or to delay treatment until the next day when there is a greater range of treatment options and their attendance time may fall outside of this study’s inclusion criteria. The study cannot quantify how frequently such options are exploited by those injured in assaults; although they are unlikely to be options for those requiring immediate attention.

In ED data, reason for attendance was coded as unknown in 4.7% of cases but data coding relies on patients revealing that their injuries have been sustained through violence and this being accurately coded in busy EDs. Although the absence of any violence related presentations from nine providers suggests under-recording of assaults, currently it is not possible to quantify the scale of such miscoding across all EDs. These issues will inevitably affect any calculation of rates. However, our findings focus on comparative risks; largely between different days or different demographics. We are not aware of any calendar, deprivation, or age/sex related bias in missing data that could confound our results, although this cannot be entirely discounted. Our focus has been on levels of emergency presentations for assault and therefore we have excluded ED attendances for follow ups relating to a previous ED attendance. We have not attempted to remove multiple presentations by the same individual for different assaults (see methods). Consequently, demographic analyses relate to probabilities of presentations being from a particular demographic. However, across the three-year study period only 6.7% of individuals presented for nighttime assault more than once and analysis of individuals, rather than presentations, would be unlikely to substantively affect results.

Sporting events included were a convenience selection based on those best known and highly promoted. There are a wide range of other local events that might have been included in this analysis and the impact of even national events (such as a football cup final) may vary with locality; if, for instance, a local team are involved. The analyses undertaken should be considered a proof of concept for the utility of ED data, which could be implemented much more widely with a complete national data set. We could not distinguish assault locations (e.g., home or city centre bar), and thus we have made no assumptions about whether assaults took place in public places or private residences. While the national ED system does not currently collect location of assault, the collection and sharing of such data at local level is increasing [11,12,57]. Finally, while this study has examined the utility of a national ED dataset in measuring calendar and demographic risk factors for nighttime assaults further analyses are now possible. ED data allows additional exploration of the residence of those involved in violence (e.g., by population density, urban vs. rural locality, etc.). Data on alcohol consumption by those presenting to EDs is not currently available nationally but, routinely collected even from a subset of EDs, could provide important intelligence on the impact of alcohol on nightlife assaults [11].

## Conclusions

Globally, national routine data collection from EDs is rare. However, it provides novel intelligence for public health. A national perspective helps avoid displacement issues [58] when assessing whether violence levels have fallen or simply moved elsewhere (e.g. a neighbouring city). ED data on nighttime assaults provide residence information and consequently, measures of socio-economic status (e.g., IMD) as well as the ability to apply population denominators for identification of rates and risk factors (e.g., by age and sex).

Risk of involvement in violence is a composite of at least environment (e.g., city centre management, access to alcohol), other proximal factors specific to the individual (e.g., employment status), and a propensity for violence that can be rooted in early childhood experiences. In these respects it has the same complex origins as other major threats to health such as obesity [59]. However, until recently the role of health services in the prevention of violence has been largely passive; with active elements limited to dealing with the physical and mental health consequences of assaults and abuse. Use of ED data, for instance, has often focused on helping target police and other regulatory activity rather than been considered as a tool to direct health interventions. This study shows how ED data might be utilised to inform frontline responses, including by EDs themselves. More importantly however, it should be central to a multiagency life course approach to the prevention of violence. A national ED system can describe the problem, identify risk and protective factors, and target prevention and protection interventions as well as assess their impact. While criminal justice systems work to contain a culture where celebrations, sports events, and holidays lead to greater violence, health services could help create one where they are not inextricably linked.

## Abbreviations

AOR: Adjusted odds ratio; CIs: Confidence intervals; DRR: Deprivation rate ratio; ED: Emergency department; GLM: Generalised linear modelling; HES: Hospital Episode Statistics; IMD: Index of Multiple Deprivation; LR: Logistic regression; LSOA: Lower super output area.

## Competing interests

The authors declare that they have no competing interests.

## Authors’ contributions

MAB designed the study and oversaw its implementation. NL, SW, ZQ undertook the data extraction, formatting and quality assurance. MAB, KH and NL analysed the data. All authors contributed to the writing of the manuscript, reviewed the study findings, read and approved the final version before submission. All authors had full access to all of the data (including statistical reports and tables) in the study and can take responsibility for the integrity of the data and the accuracy of the data analysis. MAB is the study guarantor. All authors read and approved the final manuscript.

## Pre-publication history

The pre-publication history for this paper can be accessed here:

http://www.biomedcentral.com/1471-2458/12/746/prepub
